# Investigation on the size and percentage effects of magnesium nanoparticles on thermophysical properties of reinforced calcium phosphate bone cement by molecular dynamic simulation

**DOI:** 10.1016/j.heliyon.2023.e18835

**Published:** 2023-08-02

**Authors:** Mostafa Mahjoory, Mohamad Shahgholi, Arash Karimipour

**Affiliations:** Department of Mechanical Engineering, Najafabad Branch, Islamic Azad University, Najafabad, Iran

**Keywords:** Molecular dynamics simulation, Calcium phosphate cement, Thermophysical behavior, Young's modulus, Maximum temperature

## Abstract

In recent years, bone materials and cement innovation have made extraordinary strides. Calcium phosphate cement (CPC) regenerates body tissues and repairs bone and dental defects. Since the presence of nanoparticles (NPs) increased the initial cement strength in terms of the reduction of porosity, magnesium (Mg) NPs were used because of their unique properties. In this study, the effects of various Mg NP percentages and sizes on reinforced cement thermal behavior and mechanical behavior are investigated using the molecular dynamics (MD) simulation method. The changes of Young's modulus (YM), maximum temperature (MT), and ultimate strength (US) were investigated for this reason. The US, YM, and MT of the reinforced cement sample improved from 0.879 to 0.171 MPa to 1.326 and 0.255 MPa, respectively, and from 1321 to 1403 K by raising the NPs percentage to 4%. The radius increase of NPs to 16 Å enhanced the US, YM, and MT to 0.899 MPa, 0.179 MPa, and 1349 K. The MT decreased to 1275 K. The quantity and size of the Mg NPs significantly enhanced the mechanical behavior of the finished cement, according to the findings.

## 1 Introduction

A group of bioceramics known as calcium phosphates offered a variety of medical uses, such as the treatment of bone and dental defects [1]. CPCs are a group of biomaterials that come in a variety of morphologies, have strong biodegradability, and have several uses [2,3]. The initial strength of the cement was a key factor in determining whether the body will accept an implant, and studies showed that adding NPs (nanoscale particles) increased the cement's initial strength by reducing porosity (NPs serve as cement fillers) [4]. For example, Ding et al. [1] showed the CPC's mechanical properties, stability, and strength in the presence of glycidyl methacrylate modified γ-polyglutamic acid (m-PGA) enhanced compared to the initial composite. Shojaei et al. [2] investigated the CPCs/zinc (zinc NPs) as a new and hopeful cement combination for bone repair and recovery in terms of its appropriate attributes, and amazing tissue compatibility within the body. Mg is a bone growth element that participates in bone crystal formation and strengthens bones [4]. Yoshizawa et al. [5] studied the mechanism of Mg increase in bone strength in the human body. The advantages of Mg ion NPs include non-toxicity, high thermal stability, and environmentally friendly, affordable, and unparalleled mechanical attributes, making them appropriate candidates to be used as cement fillers [6]. Zhang et al. [7] showed that the Mg nanocomposite hydrogel's stability and strength increased due to Mg ion NPs, which made it applicable in repairing bone defects.

To obtain materials mechanical properties, various approaches have been used. The most common methods are molecular dynamic simulations [8], neural network and finite element analysis [9,10], statistical estimation [11,12], numerical estimation of characteristics [13,14], different kinds of modeling [15,16] and the studies based on the experiments [17,18]. Adding NPs to the materials is a way to improve the mechanical and thermal properties of the materials or even nanofluids [19,20]. Mahjoory et al. [21] studied improving CPC's mechanical attributes by adding Mg NPs for bone repair applications. Their observations demonstrate the effect of the initial temperature and NPs percentage on CPC's mechanical properties. When Wang et al. [22] added bio-mineralized carbon nanotubes (CNTs) to the CPC, the CPC's pushing resistance increased by 24% and 120%. The 98%wt alpha tricalcium phosphate, 2%wt hydroxyapatite, and NaH_2_PO_4_ (2.5 wt%) solutions were used by Roozbahani et al. [23]. They demonstrated improved composite qualities that may be used to fill bone defects.

Biocompatible metal has clinical applications [[24], [25], [26]]. Mg and its alloys have the same density as human bones and sufficient mechanical strength to facilitate bone repair [26]. Mg has an elastic modulus of 40 GPa, which places it closer to compressed bone (23 to 30 GPa) [27] than other metals used for musculoskeletal implants, such as titanium (114 GPa) [28].

The mechanical properties of the CPCs matrix required to be described in full detail, according to Paknahad et al. [28], to improve the process of CPC properties reinforcement. To explore the dynamic aspects of the NPs system, the MD simulation approach was used [29].

Due to time and cost saving, low risk, and also a molecular investigation of the process, the MD simulation method was used in this research. In the MD simulation method, particles are allowed to interact with each other in a certain period under the well-known laws of classical physics (Newton's laws) so that a vision of particle movement is obtained [21]. Because molecular systems contain a large number of particles, it is not possible to obtain the properties of complex systems analytically. MD simulation solves this problem by using computational methods. This method creates an interface between experimental tests and theories and is considered a virtual laboratory and investigates the relationships between the structure, motion, and functions of particles [8,21]. On the other hand, according to the articles, the mechanical properties of CPC are low. In this research, the addition of Mg NPs to calcium phosphate is investigated to increase its mechanical and thermal properties. In this study, the effects of Mg percentage (1%, 2%, 3%, 4%, 5%, 6%, and 7%) and size (10 Å, 12 Å, 14 Å, and 16 Å) added to the initial matrix beside the effects of porosity (1%, 2%, 3%, and 5%) on the thermal and mechanical behavior of simulated sample were examined. In the upcoming study, the hypotheses were considered to reduce the computational cost and increase the project implementation rate. The primary structure was considered ideal and without defects from the atomic point of view. The initial temperature and pressure were assumed to be uniform via the simulation. Atomic fluctuations in the simulated ceramic structure are assumed to be small. The number of atoms was assumed to be constant.

## 2 Simulation

### 2.1 MD method

Newton's second law (Eq. (1)) established the following direct relation between the motion of a particle and the applied force [30]:

(1) $$F_{i}=m_{i}a_{i}=-\nabla_{i}U=-\frac{dU}{dr_{i}}$$

As a consequence, a method known as velocity-Verlet integration (Eqs. (2), (3))), whose algorithm is outlined as follows, is used to integrate the motion equations [31,32]:

(2) $$r_{i}\;\left(t+\Delta t\right)=2\;r_{i}\left(t\right)-r_{i}\left(t-\Delta t\right)+\left(\frac{d^{2}r_{i}}{dt^{2}}\right)\;{\left(\Delta t\right)}^{2}$$

(3) $$v\left(t+\Delta t\right)=v\left(t\right)+\Delta t\;v\left(t\right)+\frac{\Delta t\;\left(a\;\left(t\right)+a\left(t+\Delta t\right)\right)}{2}$$

EAM potential is a sum of the pairing interatomic energy ($\varphi \left(r_{ij}\right)$) between a pair of interacting atoms (i and j) and the embedding function potentials ($F\left(\rho_{i}\right)$), which are defined as Eqs. (4), (5)):

(4) $$U_{i}=\sum_{i=1}^{N-1}\sum_{j=i+1}^{N}\varphi \left(r_{ij}\right)+\sum_{i=1}^{N}F\left(\rho_{i}\right)$$

(5) $$\rho_{i}=\sum_{i=1}^{N}\psi \left(r_{ij}\right)$$

Lennard-Jones (LJ) potential approximates the potential energy of non-electrostatic interaction among a pair of non-bonded atoms or molecules with a simple mathematical function which is defined as Eq. (6) [25]:

(6) $$U_{LJ}=4\varepsilon_{ij}\left[{\left(\frac{\sigma_{ij}}{r}\right)}^{12}-{\left(\frac{\sigma_{ij}}{r}\right)}^{6}\right]$$

The values of *ε*_*ij*_ and *σ*_*ij*_ are determined for all of the interactions between the particles shown in the simulation box using the information in Table 1 and Eqs. (7), (8)) [26].

(7) $$\varepsilon_{ij}=\sqrt{\varepsilon_{i}\varepsilon_{j}}$$

(8) $$\sigma_{ij}=\frac{\sigma_{i}+\sigma_{j}}{2}$$

**Table 1:** The LJ potential function factors of NPs [26].

Particles	σ_ij_ (Å)	ε_ij_ (kcal/mol)
Ca	3.399	0.238
P	4.147	0.3050
Mg	3.021	0.111

### 2.2 Simulation details

**Fig. 1:**
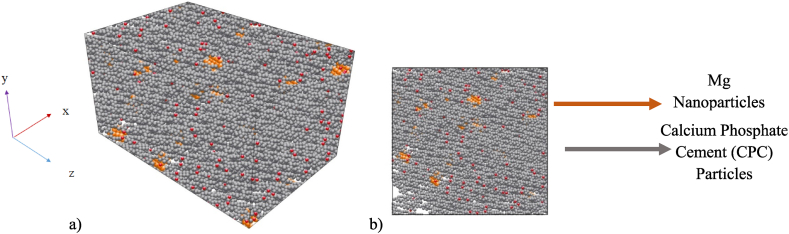
Schematic of CPC with Mg NPs.

Mg NPs were modeled homogeneous in structure. The homogeneous distribution of NPs can lead to a more uniform cement matrix reinforcement, improving its mechanical properties. However, if the NPs were clustered or unevenly distributed, this can create defects and weak points in the cement structure, reducing mechanical properties. During MD simulation, the distribution of NPs can be controlled by adjusting their initial positions and velocities. Fig. 1 (a,b) shows the schematic of CPC with Mg NPs in the first step of the MD simulation.

The atomic ratio of NPs was examined based on the number of particles inside the simulation box. Using Avogadro software, NPs can be modeled with specific numbers and desired radii. Finally, using the delete atom command, porosity was created in the simulated structure using LAMMPS software [33].

### 2.3 The equilibration process

**Fig. 2:**
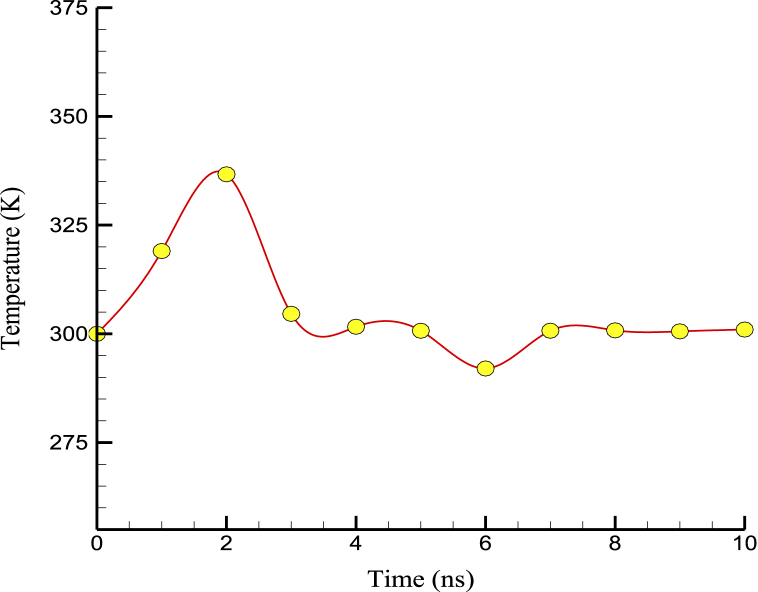
Temperature convergence in terms of simulation time.

The initial increase in temperature observed was likely since the system starts in a low-energy state, with atoms and molecules moving slowly and with low kinetic energy. As the simulation progressed and energy was added to the system, the temperature increased until it reached a maximum value. The temperature decreased after reaching this maximum value as the system equilibrated and approached a stable state. This temperature decrease was because the energy added to the system was being distributed more evenly among the atoms and molecules, leading to a more uniform distribution of kinetic energy and a decrease in temperature. The final temperature at which the system stabilizes depends on various factors, such as the initial conditions of the simulation, the parameters used to control temperature and pressure, and the specific properties of the system being simulated. In general, the final temperature should be close to the desired temperature for the simulation, typically set to a value representative of the experimental conditions or theoretical predictions. Fig. 2 shows the temperature convergence in terms of simulation time. As time advances, the temperature of structures in the examined simulation converges to a specific value, following the temperature variations of atomic structures. In general, the equilibration section of an MD simulation was an important step in ensuring that the system was stable before data collection began. By gradually adjusting the temperature and pressure, researchers can ensure that the system is at equilibrium and that any subsequent results are reliable and accurate.

**Fig. 3:**
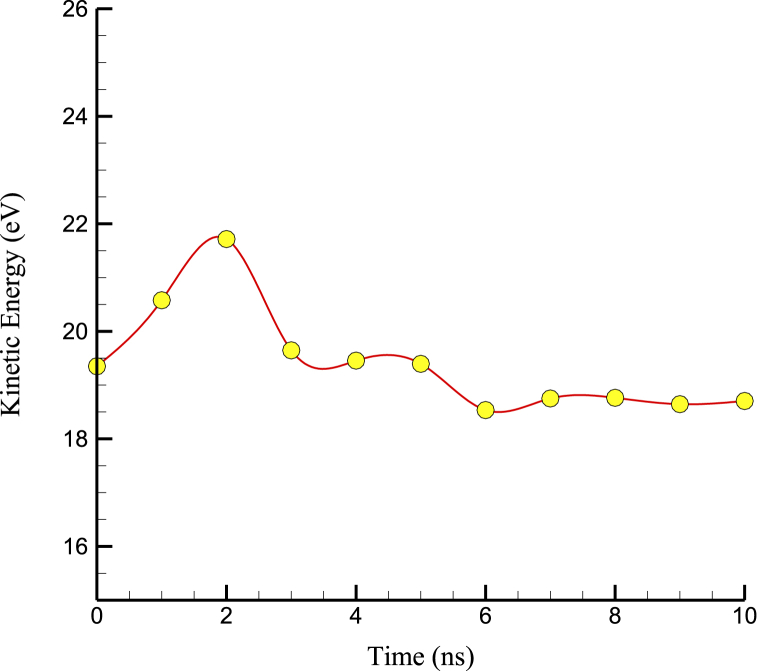
Kinetic energy convergence in terms of simulation time.

The kinetic energy of simulated structures, on the other hand, was proportional to their temperature because the atomic oscillations and temperature in these structures were proportional to the mobility and movement of the particles. As a result, with the convergence of temperature in the atomic structures, the kinetic energy also converged. Kinetic energy convergence in terms of simulation time is shown in Fig. 3. This behavior is due to the atomic velocity's proportionality to the simulated samples' temperature.

## 3 Results and discussion

Physical quantities including stress, YM, US, and MT were recorded and studied in this study. Nose-Hoover and NVT thermostats were used to raise the temperature technically.

### 3.1 The effect of Mg NPs percentage

**Fig. 4:**
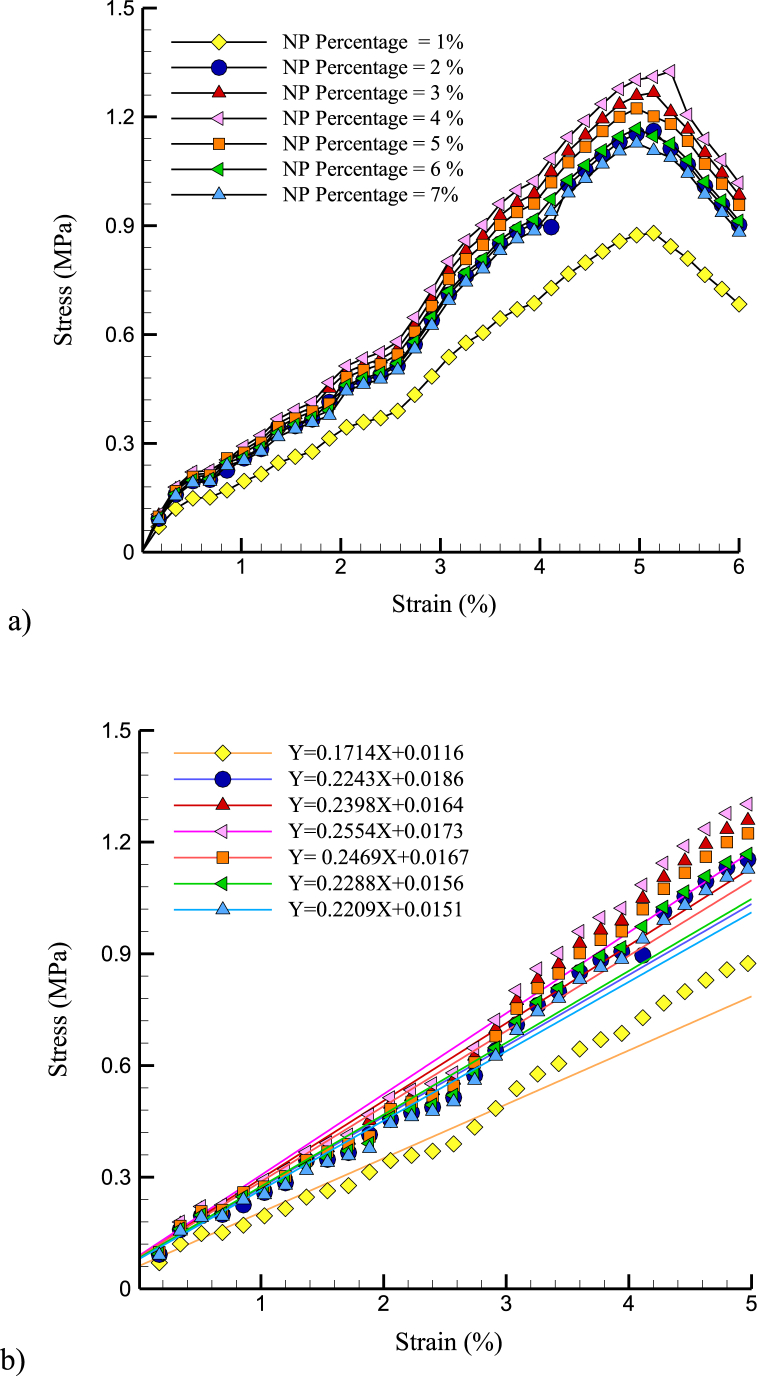
a) The stress-strain and b) YM variations over the NP percentage.

Fig. 4 (a, b) shows the stress-strain and YM variations over the NP percentage. According to increasing Mg NPs added, the stress-strain of samples is shown in Fig. 4. According to Fig. 4, the mechanical behavior of structures approached their ideal condition when the NP percentage in the atomic samples increased to 4%.

**Fig. 5:**
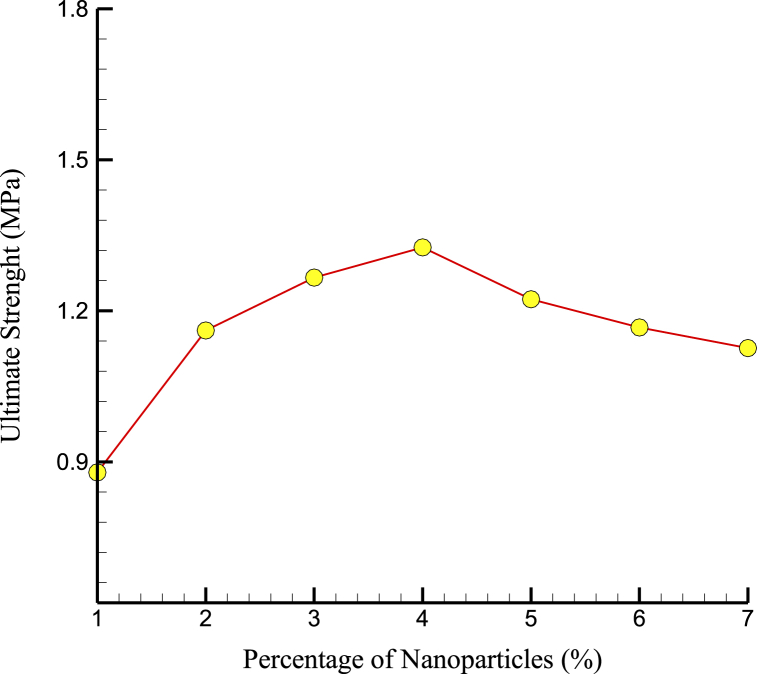
The US variations over NPs percentage.

**Fig. 6:**
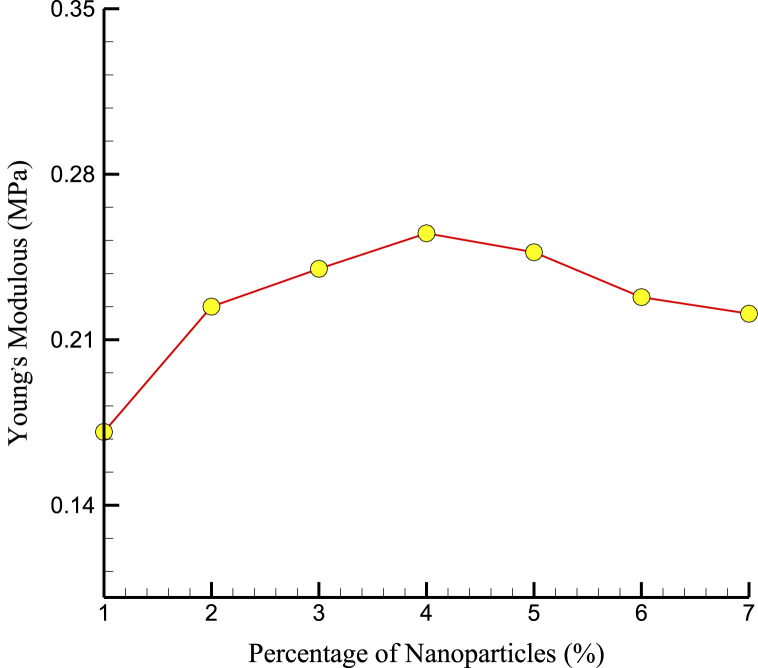
YM variations per percentage of NPs after 10 ns.

Fig. 5, Fig. 6 show the US and YM variations over NPs percentage. An ideal density of NPs may provide the most efficient reinforcing of the cement matrix at 4% NP concentration. NPs may begin to agglomerate or cluster at higher NP concentrations, creating defects and weak points in the cement structure that reduce its mechanical properties. Furthermore, the reduction in interatomic force and mechanical strength at higher NP percentages may be because the NPs were too densely packed within the matrix. At higher NP concentrations, the NPs may begin to compete for space within the matrix, leading to reduced interatomic forces and mechanical strength. The mechanical properties of bone, such as strength and stiffness, were important factors that determine its ability to support weight and resist fracture. The mechanical properties of CPC with added Mg NPs might relate to the mechanical properties of bone in several ways. For example, bone contains mineral components, such as hydroxyapatite, contributing to its stiffness and strength. Adding Mg NPs to CPC may enhance the mineral content of the material, leading to improved mechanical properties that were more similar to those of bone.

**Fig. 7:**
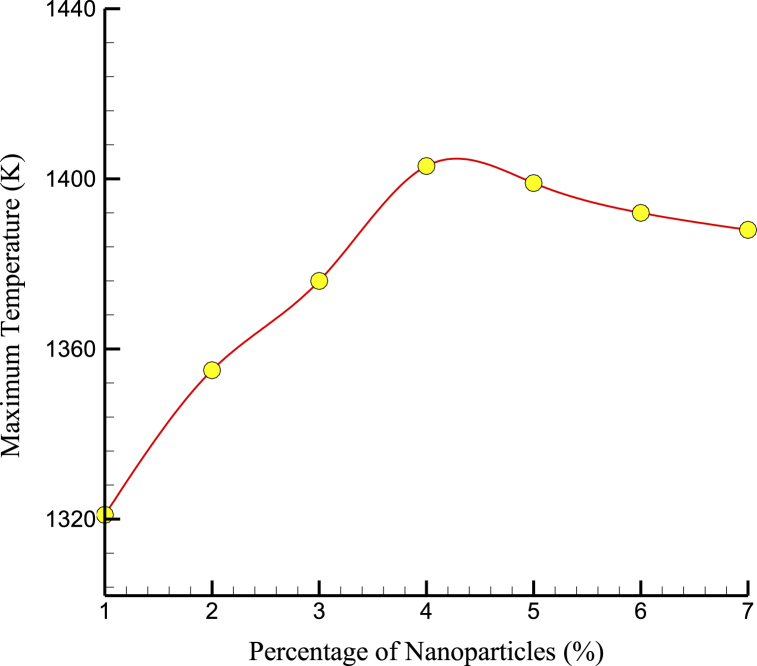
The MT variations over the NPs percentage.

Fig. 7 shows the MT MT variations over the NPs percentage. The greatest stability temperature in the improved matrix sample was 1403 K after adding 4% of NPs to the initial matrix. However, beyond 4% NP concentration, the MT decreased with a further increase in NP concentration. This is likely because the NPs began to agglomerate or cluster at higher NP concentrations, creating defects and weak points in the cement structure. These defects and weak points can reduce the material's mechanical properties and lead to a decrease in the energy absorbed by the system during loading, resulting in a lower MT. Thermophysical quantities of CPC in terms of percentage of NPs are shown in Table 2.

**Table 2:** Thermophysical quantities of CPC in terms of percentage of NPs.

NPs Percentage (%)	US (MPa)	YM (MPa)	MT (K)
1	0.879	0.171	1321
2	1.161	0.224	1355
3	1.266	0.240	1376
4	1.326	0.255	1403
5	1.223	0.247	1399
6	1.167	0.228	1392
7	1.126	0.221	1388

### 3.2 The effect of Mg NPs size

**Fig. 8:**
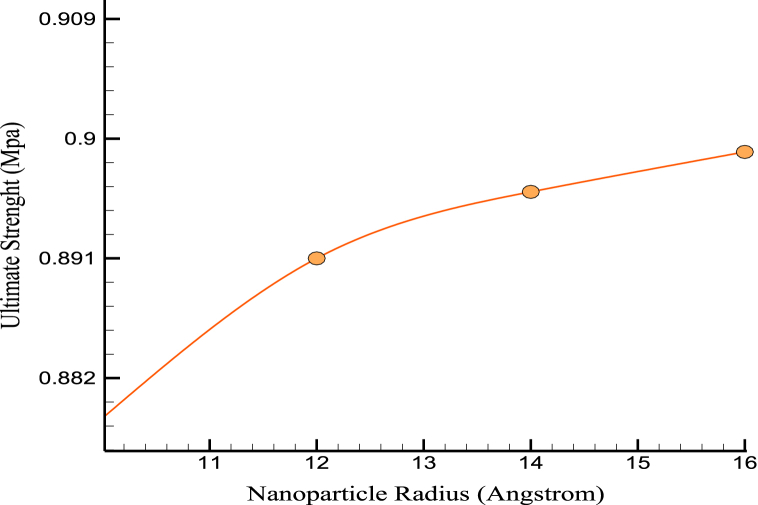
The changes of US over the NPs' radius.

**Fig. 9:**
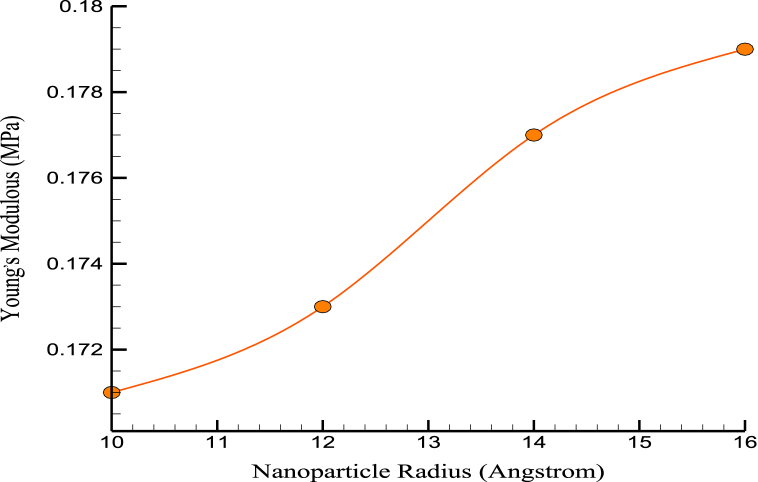
YM variations over the NPs' radius.

Based on mechanical findings, it was found that the simulated structures' stress-strain relationships and mechanical behavior remained mostly unchanged. Fig. 8, Fig. 9 show the US and YM variations over the NPs' radius. The size of Mg NPs can significantly affect the mechanical properties of CPC, and how they relate to the mechanical properties of bone. MD simulations can provide insights into the behavior of Mg NPs in CPC, and how they affect the material's mechanical properties. For example, simulations can show how Mg NPs interact with the surrounding cement matrix and how this affects the formation and growth of the cement structure. Larger NPs can reinforce the cement matrix more effectively, improving load-bearing capacity. However, there is a limit to how large Mg NPs can be before they negatively affect the material's mechanical properties. Large NPs can create defects and discontinuities in the cement structure, reducing strength and stiffness. In terms of how these mechanical property simulations relate to the mechanical properties of bone, bone is a highly complex and heterogeneous material with a complex structure that includes mineral and organic components. While adding Mg NPs to CPC can improve its mechanical properties, it is unlikely to replicate the complex structure and properties of bone fully. However, using Mg NPs in CPC may help improve its biocompatibility and promote bone growth and remodeling, which are important factors in bone repair and regeneration.

**Fig. 10:**
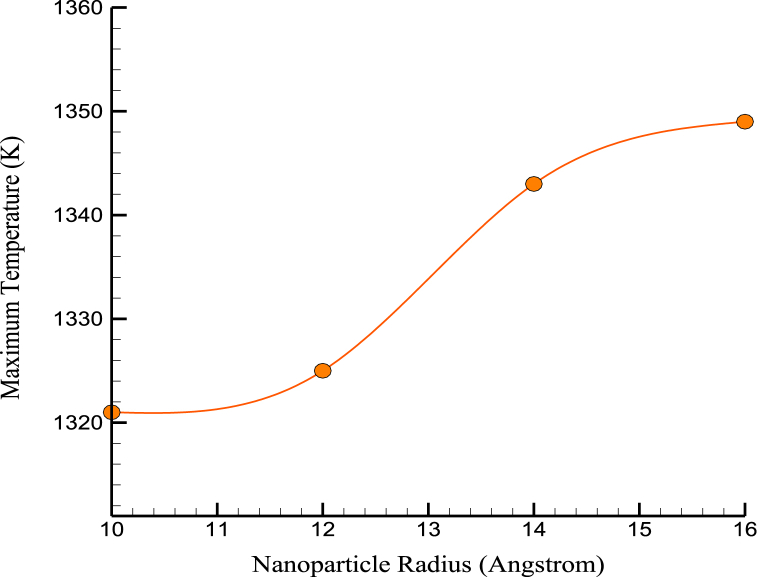
The changes of MT over the NPs' radius.

Fig. 10 shows the changes in MT over the NP's radius. According to numerical calculations using the average radius of the NPs that were added to the original cement, the system's MT at which atomic stability was maintained was 1349 K. This characteristic of enhanced matrices improved the cement's performance at high temperatures. Thermophysical quantities of CPC in terms of NPs' radius are reported in Table 3.

**Table 3:** Thermophysical quantities of CPC in terms of NPs’ radius.

NPs Radius (Å)	US (MPa)	YM (MPa)	MT (K)
10	0.879	0.171	1321
12	0.891	0.173	1325
14	0.896	0.177	1343
16	0.899	0.179	1349

### 3.3 The effect of porosity

**Fig. 11:**
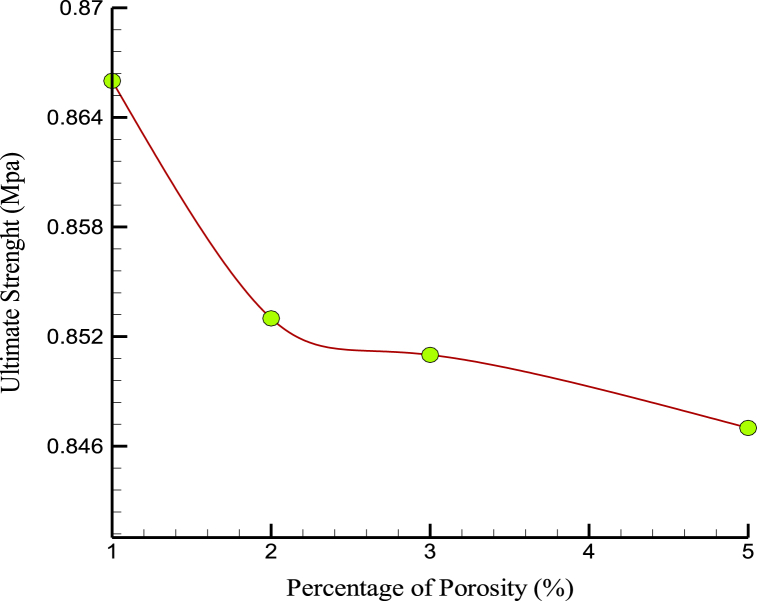
The changes in the US over the percentage of porosity.

**Fig. 12:**
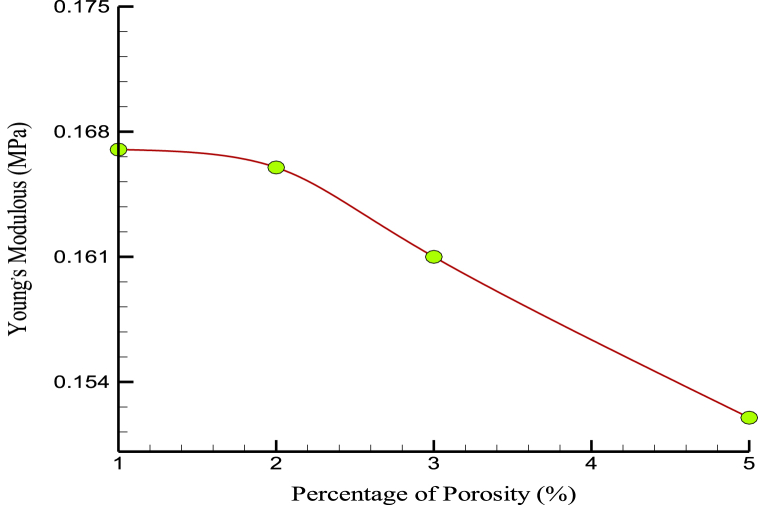
YM variations over the percentage of porosity.

This factor can be caused by atomic oscillation in the different samples, temperature fluctuations, and unintentional. On the other hand, in some production processes, atomic porosity is created to reduce the weight of the samples. Fig. 11, Fig. 12 show the changes in US and YM over the percentage of porosity. Atomic porosity, or the presence of voids or empty spaces at the atomic level, can significantly affect. MD results suggest that increasing atomic porosity in CPC can decrease its US and YM. This is because voids or empty spaces can weaken the cement structure and reduce its load-bearing capacity. In bone, atomic porosity is an important factor influencing its mechanical properties. Bone contains complex mineral and organic components, including microscopic pores and channels. These pores and channels are important in bone's ability to absorb and distribute mechanical loads. However, excessive porosity in the bone can lead to reduced mechanical properties, such as decreased stiffness and strength. This is because voids or empty spaces can weaken the bone structure and reduce its load-bearing capacity. While some degree of atomic porosity is important for the mechanical properties of CPC and bone, excessive porosity can negatively affect their strength and stiffness. Therefore, it is important to carefully control the porosity of these materials to optimize their mechanical properties for specific applications.

**Fig. 13:**
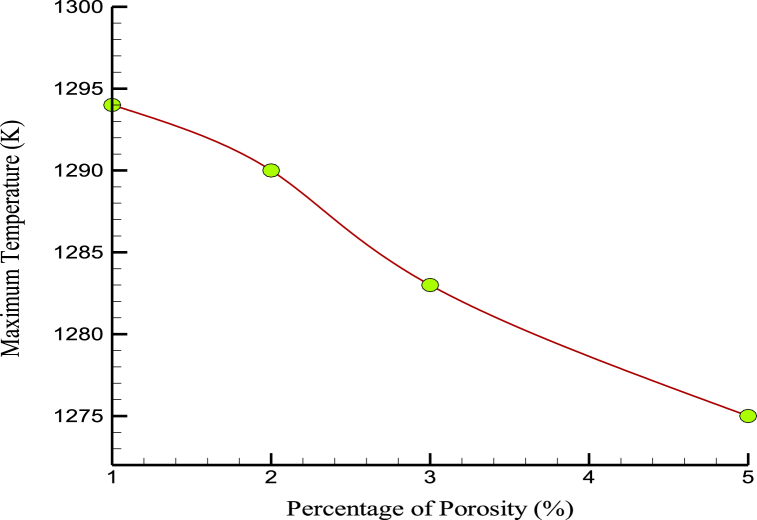
The MT variations over the percentage of porosity.

The mobility and kinetic energy of atoms in a simulated box were increased by temperature change (temperature increase) through porosity in the prescribed structures. Fig. 13 shows the changes in the amplified matrix's MT with changes in the initial sample's simulated porosity. Numerically, the MT in the samples decreased to 1275 K when the porosity in the atomic samples was increased. This should be taken into account when using developed nanocomposites in industry and medicine. Thermophysical quantities of CPC in terms of porosity are reported in Table 4.

**Table 4:** Thermophysical quantities of CPC in terms of porosity.

Porosity (%)	US (MPa)	YM (MPa)	MT (K)
1	0.866	0.167	1294
2	0.853	0.166	1290
3	0.851	0.161	1283
5	0.847	0.152	1275

## 4 Conclusion

This work studied the thermal and mechanical behaviors of CPCs reinforced with Mg NPs using MD simulation procedure. The numerically obtained results are as follows:

• By increasing the atomic percentage of MG NPs up to 4%, the US and YM increased from 0.879 to 0.171 MPa to 1.326 and 0.255 MPa.
• Moreover, increasing the atomic percentage of MG NPs up to 4% enhanced the MT to 1403 K.
• As the percentage of porosity increased from 1%wt to 5%wt, the US, YM, and MT reduced from 0.866, 0.167 MPa, and 1294 K to 0.847, 0.152 MPa, and 1275 K, respectively.

One of the limitations of the topic under discussion is high laboratory costs and animal and clinical tests. Also, the patient's body may show an allergic reaction due to the substances used instead of the bones. The polymerization reaction takes place at the junction of the bones of the body and produces a lot of heat, which raises the temperature of that area to approximately 80–120 °C. This temperature destroys the soft tissue around the bones and also destroys part of the bone marrow. This destruction of tissues affects the mechanical function of the body over time and causes a person to feel pain while moving their organs. After long-term use of these cement in the body, the mechanical properties are reduced, which must be replaced.

### 4.1 Practical application

The results of MD simulations can then be used to guide experimental efforts, such as optimizing the synthesis process or testing new formulations of the cement. By combining MD simulation with experimental methods, the researchers can accelerate the development of new materials with improved properties for various applications. MD results, such as atomic-level details of the structure and bonding of the cement particles, can help researchers understand how to optimize the synthesis process to produce stronger and more stable cement. Or information on the mechanical properties of the cement, such as its stiffness, strength, and fracture toughness. This can help researchers identify ways to improve the mechanical properties of the cement, which is important for applications such as bone repair and dental implants. The insights into the behavior of cement in different environments, such as in contact with biological tissues or in the presence of different ions. This information can help researchers design more biocompatible cement with better integration with surrounding tissues. Moreover, the predictions of long-term stability and degradation of the cement are important for applications where it needs to maintain its properties over extended periods, such as in orthopedic implants. In general, MD simulation can provide a wealth of physical results that can help researchers optimize the properties of CPC and accelerate the development of new materials for a wide range of applications.

### 4.2 Further research

To study the mechanical and thermal behavior of improved cement matrix samples as much as possible, it is suggested to use composite NPs in atomic samples and different shapes and the effect of increasing and decreasing temperature and pressure on mechanical and thermal behavior.

## Author contribution statement

Mostafa Mahjoory: Contributed analysis tools or data, wrote the paper;

Mohamad Shahgholi: Analyzed and interpreted the data;

Arash Karimipour: Conceived and designed the analysis;

## Data availability statement

Data will be made available on request.

## Declaration of competing interest

The authors declare that they have no known competing financial interests or personal relationships that could have appeared to influence the work reported in this paper.
